# 

**Published:** 1922-11

**Authors:** 


### HOSPITAL and HEALTH

REVIEW

Established 1886 No. 1856. Vol. LXXII.

New Series. Vol. II. No. 14 November 1922 Price Sixpence

CONTENTS.

| | PAGE |
|---|---|
| NOTES AND COMMENTS— | |
| Surmounting the Hospital Crisis—The Outside of the Tin—Botulism—Ex-Service Men in Asylums—Rapid Fall of the Tuberculosis Death-rate in New York—Education at the Cost of Health—The New Elixir of Life—A New Disease—Holiday Centres and Epidemics—The Bolshevist Blight—The Dangers of Hair Dyes—Jenner Anticipated—Motor Accidents—The Fumigation of Ships—Liquid Eggs—The Fathers' Committee—Euthanasia—Labour Hospitals in Belgium—The Poor Law Patient's Right to Discharge—Doctors and the County Hospitals—The Co-ordination Movement—A Hospital's Protest . . . . . | 1 |
| THE PUBLIC HEALTH: INTERVIEWS WITH LOCAL AUTHORITIES. V.—NOTTINGHAM: AN INDUSTRIAL HEALTH RESORT . . . . . . | 9 |
| MEDICINES AND MEDDLESOMENESS.—II. . . . By R. W. Harris. | 12 |
| NATIONAL HEALTH INSURANCE NOTES AND COMMENTS | 13 |
| MILK . . . . . . . . . . . . | 15 |
| The Administration of the Law Relating to Milk | |
| Pure and Clean Milk | |
| THE MILK SUPPLY IN AMERICA . . . . . | 16 |
| THE STERILISATION OF UNDESIRABLE CITIZENS . . . By Col. G. T. K. Maurice, C.M.G. | 17 |
| THE HEALTH OF THE SCHOOL CHILD . . . . | 17 |
| MOSQUITOES AND MALARIA IN ENGLAND . . . By Col. S. P. James. | 19 |
| THE CONTROL OF HOSPITAL EXPENDITURE: THE BUDGET OR ESTIMATE SYSTEM . . . . . | 20 |
| By Joseph E. Stone, F.S.A.A., F.S.S., F.R.Econ.S. | |
| THE FUTURE OF HOSPITALS . . . . . . | 21 |
| HOSPITAL FINANCE . . . . . . . | 22 |
| RECENT APPOINTMENTS . . . . . . | 24 |
| GOVERNMENT PUBLICATIONS . . . . . | 24 |
| OFFICIAL REPORTS RECEIVED . . . . . | 24 |
| HOSPITAL NEWS . . . . . . . | 25 |
| NURSING IN INDIA . . . . . . . . | 26 |
| AN OPEN-AIR HOSPITAL WARD . . . . . | 27 |
| REVIEWS . . . . . . . . . | 28 |
| CORRESPONDENCE— | |
| Home Nursing for Insured Persons . . . . | 31 |
| THE NATIONAL INSTITUTE FOR THE BLIND . . . . | 32 |

